# Effects of pirfenidone targeting the tumor microenvironment and tumor-stroma interaction as a novel treatment for non-small cell lung cancer

**DOI:** 10.1038/s41598-020-67904-8

**Published:** 2020-07-02

**Authors:** Ayako Fujiwara, Soichiro Funaki, Eriko Fukui, Kenji Kimura, Takashi Kanou, Naoko Ose, Masato Minami, Yasushi Shintani

**Affiliations:** Department of General Thoracic Surgery, Osaka University Graduate School of Medicine, 2-2-L5 Yamadaoka, Suita, Osaka 565-0871 Japan

**Keywords:** Cancer microenvironment, Lung cancer

## Abstract

Targeting cancer-associated fibroblasts (CAFs), as well as the crosstalk between stroma and cancer cells, could be of value in managing cancers. Pirfenidone (PFD) is an anti-fibrotic agent for idiopathic pulmonary fibrosis. This study aimed to investigate the possibility that PFD might exert an anti-tumor effect through inhibition of fibroblast activation and the tumor-stroma interaction in non-small cell lung cancer (NSCLC) cell lines in vitro and in vivo. PFD significantly inhibited myofibroblast differentiation and activation of both primary cultured normal human lung fibroblasts and CAFs. Cocultivation of NSCLC cells with conditioned media (CM) of fibroblasts changed the morphology or epithelial to mesenchymal transition (EMT) status, and PFD suppressed these changes. Cocultivation of CAFs with CM of NSCLC cells also induced activation of CAFs, and these changes were suppressed by PFD. On in vivo examination, CAFs promoted tumor progression, and PFD suppressed tumor progression with an inhibitory effect on tumor-stroma crosstalk. PFD might inhibit not only fibroblast activity, but also the crosstalk between cancer cells and fibroblasts. PFD may have great potential as a novel treatment for NSCLC from multiple perspectives.

## Introduction

Mortality rates in non-small cell lung cancer (NSCLC) patients remain excessively high despite the implementation of novel targeted therapies and chemotherapeutic regimes because of the tumor’s high metastatic ability^1^. Tumors are complex structures composed of cancer cells surrounded by the tumor stroma^2^, and the tumor stroma is attracting attention because it plays a central role in tumor development, invasion, and metastasis^3^. Cancer-associated fibroblasts (CAFs) are the major constituent of the tumor stroma. They are believed to be prominent contributors to carcinogenesis, proliferation, and invasion, and they play key roles in promoting cancer progression^4^. Contact between CAFs and tumor cells has been shown to increase tumor cell survival via the activation of anti-apoptotic pathways or by induction of the epithelial to mesenchymal transition (EMT)^5,6^. Thus, targeting CAFs, as well as the crosstalk between stroma and cancer cells, could be of value in managing cancers.

Pirfenidone (PFD, Shionogi & Co., Ltd., Osaka, Japan) is a pyridine compound with therapeutic potential for idiopathic pulmonary fibrosis (IPF). In previous studies, PFD has been shown to inhibit fibrosis and/or desmoplasia in vivo, as well as proliferation and/or activation of fibroblasts in vitro in various organs and cell lines^7–9^. In addition, Jin et al. analyzed mechanisms of the lung fibroblast-dependent anti-fibrotic effects of PFD and showed that TGF-β1-stimulated bioactivity was attenuated by changing IPF lung-relevant gene expression^10^. TGF-β1 is a key mediator of normal tissue repair and strongly stimulates mesenchymal cells to produce large amounts of ECM, including fibronectin and collagen, resulting in the development of fibrosis^11^. Referring to malignancies, it was recently reported that PFD induced apoptotic cell death in lung CAFs, and it increased apoptosis and synergistic cell death when combined with cisplatin^12^. Takai et al. also reported that PFD inhibited cell viability and collagen production of CAFs, as well as the tumor growth induced by CAFs in breast cancer^13^. Polydorou et al. also reported that PFD normalized the tumor microenvironment so that it may enhance drug delivery to solid tumors using two orthotopic mammary tumor models^14^. We have also previously reported that PFD could inhibit the EMT in NSCLC cell lines through inhibiting TGF-β signaling and TGF-β production^15^. However, the effect of PFD on tumor stroma remains unclear. Since TGF-β is important for the tumor-stromal interaction^16,17^, we hypothesized that PFD may also inhibit the tumor-stroma interaction in NSCLC.

Thus, the purpose of the present study was to conduct further analyses of the effects of PFD in the tumor microenvironment of NSCLC. The effects of PFD on the characteristics of normal fibroblast cell lines, as well as clinical samples of lung normal fibroblasts (LNFs) and CAFs, were evaluated. In addition, the interactions between CAFs and NSCLC cells were examined in vitro and in vivo.

## Results

### PFD inhibited activation in LNFs and CAFs

First, normal fibroblast cell lines were used to confirm the inhibitory effect of PFD on myofibroblast differentiation and activation. Myofibroblast differentiation and activation of NHLFs by TGF-β1 were suppressed by PFD, consistent with previous work (Sup. Figure 1). To investigate the mechanism of PFD inhibition of TGF-β1-induced activation of NHLFs, its effects on TGF-β1-induced Smad phosphorylation were examined. Western blot analysis showed that the expression of phosphorylated-Smad2 was increased by TGF-β1 and inhibited by PFD. The expression of phosphorylated-Stat3 was also evaluated because it is a major downstream mediator of IL-6 receptors, and PFD decreased IL-6 production. Although the expression of phosphorylated-Stat3 was not affected by TGF-β1, as expected, it was suppressed by 500 μg/mL PFD compared with control. These data suggest that myofibroblast differentiation and activation of NHLFs by TGF-β1 were suppressed by PFD, followed by attenuation of IL-6 signaling.

**Figure 1:**
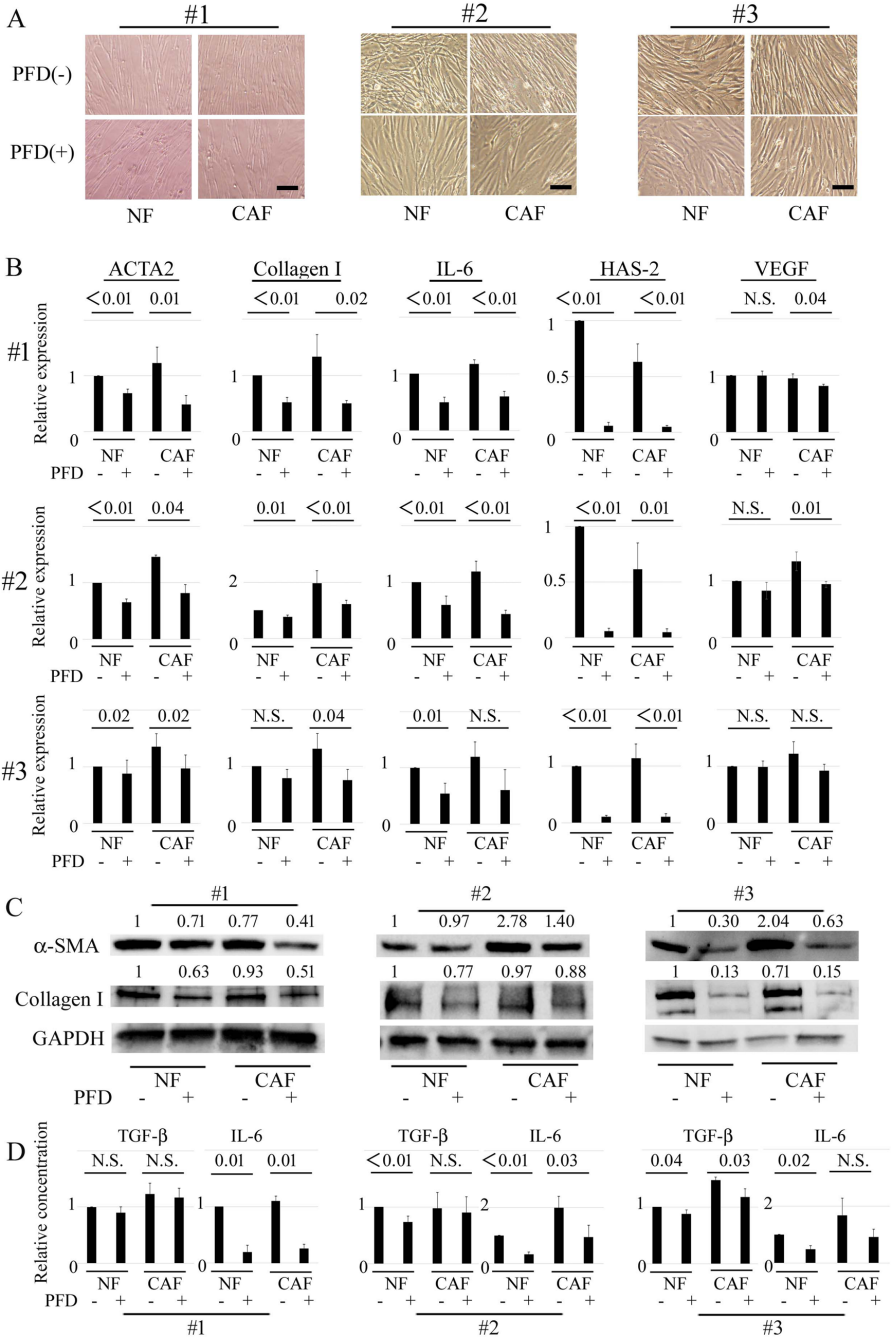
Inhibitory effects of pirfenidone on the activation of primary cultured fibroblasts. (**A)** Cancer-associated fibroblasts (CAFs) are defined as fibroblasts obtained from tumors. Lung normal fibroblasts (LNFs) are defined as fibroblasts obtained from normal lung tissue. CAFs and LNFs are treated with DMSO (control) or pirfenidone (PFD, 500 μg/mL) for 2 days. Phase contrast pictures were taken. Scale bar: 100 µm. (**B**) CAFs and LNFs are treated with DMSO or PFD for 24 h. Subsequently, total RNA is extracted, and real-time RT-PCR is performed to detect ACTA2, collagen I, IL-6, hyaluronan synthase 2, and VEGF mRNA. All experiments were performed three times independently with duplicates, and the results are the means ± SD. Significance was tested with the Mann–Whitney U test. (**C**) CAFs and LNFs are treated with DMSO or PFD for 2 days and then analyzed by Western blotting for α-SMA, collagen I, and GAPDH (loading control). All experiments were performed two times independently. Data shown are representative experiments and their quantitative values. (**D**) CAFs and LNFs are treated with DMSO or PFD for 2 days. The medium is changed to FBS-free medium, and TGF-β1 or IL-6 in the conditioned medium is then measured 24 h after the medium change using ELISA. All experiments were performed three times independently with duplicates, and the results are the means ± SD. Significance was tested with the Mann–Whitney U test.

The effects of PFD in primary cultured CAFs and LNFs were next examined. To maintain universality as much as possible, cells from different tissue types were used, with type #1 derived from an adenosquamous carcinoma, #2 from an adenocarcinoma, and #3 from a squamous cell carcinoma. Under phase-contrast microscopy, the cells showed the typical spindle-like shape of fibroblasts with no apparent difference in all cells derived from the 3 NSCLC tissues, and the slenderness seemed to be mildly suppressed by co-treatment with PFD, both in the CAFs and LNFs (Fig. 1A). The mRNA levels of ACTA2, collagen I, and IL-6 were greater in the CAFs than in the LNFs (average 1.38-fold, range 1.17- to 1.96-fold), whereas the expressions in both CAFs and LNFs were inhibited by co-treatment with PFD in all 3 NSCLC cell lines, with an average reduction rate of 0.48 and 0.63, respectively (Fig. 1B). Though the mRNA level of hyaluronic acid synthase 2 (HAS-2) was not greater in the CAFs than in the LNFs, expression was strongly inhibited by co-treatment with PFD, with an average reduction rate of 0.07 in LNFs and 0.08 in CAFs. The Western blot analysis showed the same tendency as RT-PCR for the effects of PFD (Fig. 1C).

The inhibitory effects of PFD on cytokine production were examined next (Fig. 1D). ELISA findings showed that the concentration of IL-6 was greater in CAFs than LNFs (1.64-fold), whereas the concentration of TGF-β1 was nearly equal, at only 1.09-fold greater. PFD inhibited cytokine production of both IL-6 and TGF-β1, though the inhibitory effect was greater with IL-6, with an average reduction rate of 0.65 compared to that of 0.10 for TGF-β1. These data suggest that PFD inhibited the activation level, as well as the cytokine production, of LNFs and CAFs.

### Inhibitory effects of PFD on the interaction between CAFs or LNFs and NSCLC cells

**Figure 2:**
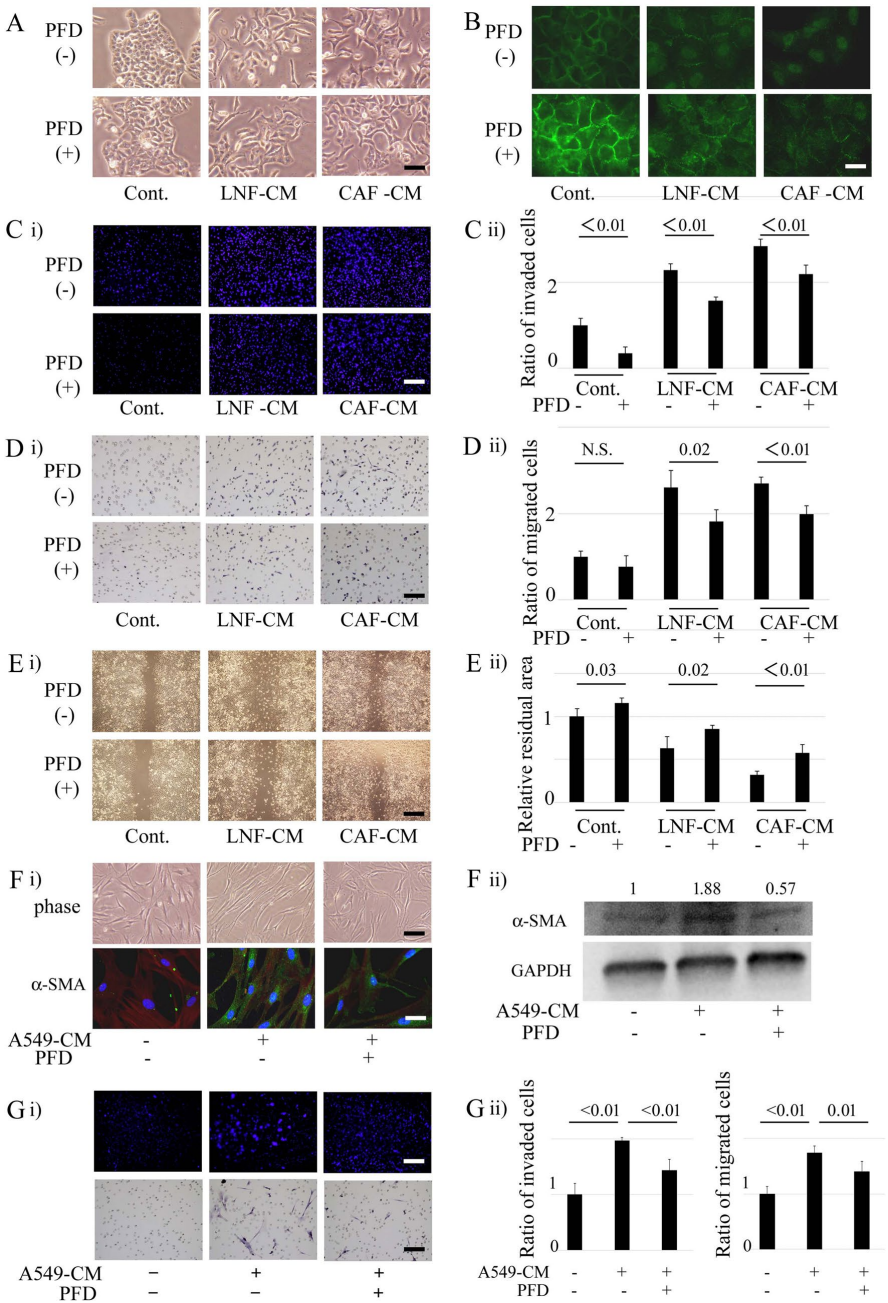
Inhibitory effects of pirfenidone on the interaction between primary cultured fibroblasts and non-small cell lung cancer cells. (**A**) A549 cells are cultured with conditioned medium (CM) from cancer-associated fibroblasts (CAFs) or lung normal fibroblasts (LNFs) for 2 days and compared to the A549 cells cultured with normal DMEM. In the process of CM preparation, CAFs or LNFs are cultured with or without pirfenidone (PFD) for 24 h, and then PFD is removed. Phase contrast pictures were taken. Scale bar: 100 µm. (**B**) A549 cells are treated as in **A**, and the cells are then immunofluorescently stained with E-cadherin. Scale bar: 50 µm. (**C**) Invasion assays are performed, and cells traversing the filter counted. DMSO, PFD (500 µg/mL), LNF-CM, or CAF-CM with or without PFD pretreatment is added to the lower side of the chamber. Panel (i) shows representative findings. Scale bar: 200 µm. Panel (ii) shows the ratio of invaded cells compared to control. All experiments were performed three times independently, and columns represent the means ± SD. (**D**) Motility assays are performed, and cells traversing the filter counted. DMSO, PFD (500 µg/mL), LNF-CM, or CAF-CM with or without PFD pretreatment is added to the lower side of the chamber. Panel (i) shows representative findings. Scale bar: 130 µm. Panel (ii) shows the ratio of invaded cells compared to control. All experiments were performed three times independently, and the columns represent the means ± SD. (**E**) Wound healing assays are performed, and the remaining area that escaped from cell migration is measured. DMSO, PFD (500 µg/mL), LNF-CM, or CAF-CM with or without PFD pretreatment is added after scratching. Panel (i) shows representative findings. Scale bar: 400 µm. Panel (ii) shows the ratio of invaded cells compared to control. All experiments were performed three times independently, and columns represent the means ± SD. (**F**) CAFs are cultured with CM from A549 cells for 2 days and compared to control. In the process of CM preparation, A549 cells are cultured with or without PFD for 24 h, and then PFD is removed. Panel (i) upper part: phase contrast pictures. Scale bar: 200 µm. lower part: immunofluorescence staining for α-SMA. Scale bar: 50 µm. Panel (ii) Treated CAFs are analyzed by Western blotting for α-SMA and GAPDH (loading control). Experiments were performed two times independently. Data shown are representative experiments and their quantitative values. (**G**) Invasion and motility assays of CAFs are performed, and cells traversing the filter counted. DMSO or A549-CM with or without PFD pretreatment is added to the lower side of the chamber. Panel (i) upper part: representative findings of invasion assays. Scale bar: 200 µm. lower part: representative findings of motility assays. Scale bar: 200 µm. Panel (ii) left part: the ratio of invaded cells compared to control. right part: the ratio of migrated cells compared to control. All experiments were performed three times independently, and the columns represent the means ± SD.

To explore the effects of PFD on the interaction of NSCLC cells and CAFs or LNFs, A549 and NCI-H358 cells were first cultured with the CM of CAFs or LNFs. To determine the effects of PFD on the interactions of these cells, not on the cells themselves, PFD was removed after 24 h of treatment in the process of CM preparation, as described in Methods. To achieve universality, A549 was treated with CM from cells #3 of Fig. 1, and NCI-H358 was treated with cells #2. Treatment with the CAFs or LNFs CM dramatically changed the morphology of A549 cells to a sharp, irregular shape with a diminished effect of cell–cell contact, and pre-treatment of CAFs or LNFs with PFD showed significant inhibitory effects on these changes (Fig. 2A). The adhesion properties of A549 cells were evaluated by immunofluorescence of the expression of the adhesion protein E-cadherin (Fig. 2B). The expression of E-cadherin was decreased at cell–cell contacts by CM of CAFs or LNFs, with a more prominent reducing effect in CAF-CM-treated cells, and PFD pretreatment restored the expression of E-cadherin in both cell lines. Whereas Western blotting showed that total E-cadherin protein expression levels were decreased in response to CM of CAFs, they were not changed by PFD (Sup. Figure 2). Fibronectin expression levels increased in response to CM of LNFs or CAFs, which were attenuated by PFD. The invasion and migration of NSCLC cells cultured with CM of CAFs or LNFs were then examined. The results showed that treatment of CAFs or LNFs with CM markedly enhanced the invasion of A549 cells compared with untreated cells, with a more prominent increase seen in CAF-CM than LNF-treated cells (increase rate 2.84 vs. 2.28) (Fig. 2C). In contrast, PFD pretreatment reduced A549 cell invasion. A549 cell migration was also increased by treatment with CM of CAF or LNF CM by 2.60 and 2.70, respectively, and inhibited by pretreatment of CAFs or LNFs with PFD (Fig. 2D). Similar results were obtained in the wound-healing assay (Fig. 2E). The residual area in the center, composed of migrated cells from both sides, indicates narrowing by treatment with CM of CAFs or LNFs. Furthermore, it was attenuated by pretreatment of CM of CAFs with PFD as compared to CM derived from non-treated cells, indicating that PFD pretreatment of CM of CAFs weakened A549 cell migration. That is, CM treatment increased the migratory potential of NSCLC cells, and PFD pretreatment of CM of CAFs or LNFs decreased the invasion and migration ability of NSCLC cells according to all of the results shown in Fig. 2C–E. Similar results were seen in experiments using NCI-H358 (Sup. Figure 3). NCI-H520 cells, a squamous cell carcinoma cell line, were also used. Whereas E-cadherin and vimentin expression levels were not changed in response to CM or PFD, fibronectin expression levels increased in response to CM of LNFs or CAFs. Fibronectin and vimentin expressions were attenuated by PFD (Sup. Figure 4). These results suggested that, whereas changes of EMT markers differed depending on cell types, PFD might affect some of those changes.

**Figure 3:**
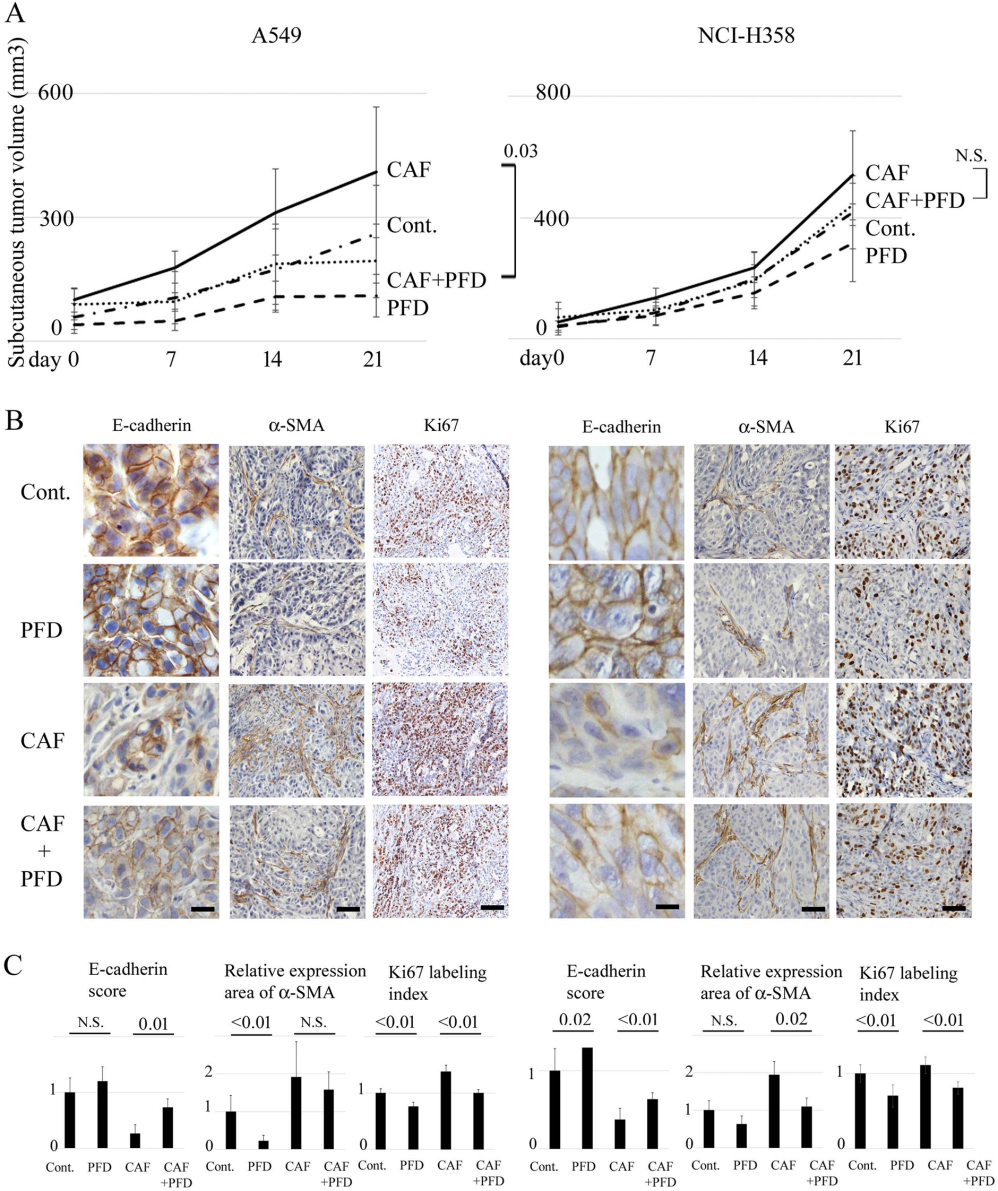
Effects of pirfenidone on subcutaneous tumor formation of NSCLC cells and CAFs in vivo. (**A**) Tumor volumes (mean ± SD) after subcutaneous injection of A549 or NCI-H358 cells with or without CAFs into nude mice are plotted, and significance tested with repeated measures ANOVA. Mice are treated intraperitoneally with 200 mg/kg pirfenidone (PFD) or sterile water daily for 3 weeks. Each group contains six mice. The chain shows the tumor volume of control mice (Cont.): NSCLC cells only are transplanted without PFD treatment. Broken line (PFD): NSCLC cells only are transplanted with PFD treatment. Solid line (CAF): both NSCLC cells and CAFs are transplanted without PFD treatment. Dotted line (CAF + PFD): both NSCLC cells and CAFs are transplanted with PFD treatment. (**B**) Panels show representative findings of histological evaluation for E-cadherin, α-SMA, and Ki67 of primary tumors obtained from mice. Scale bar: 80 µm for E-cadherin, 200 µm for α-SMA, and 400 µm for Ki67. (**C**) Panels show the scored expression of E-cadherin, the relative area of α-SMA expression, and the Ki67 labeling index as the mean ± SD. Significance was tested with the Mann–Whitney U test.

**Figure 4:**
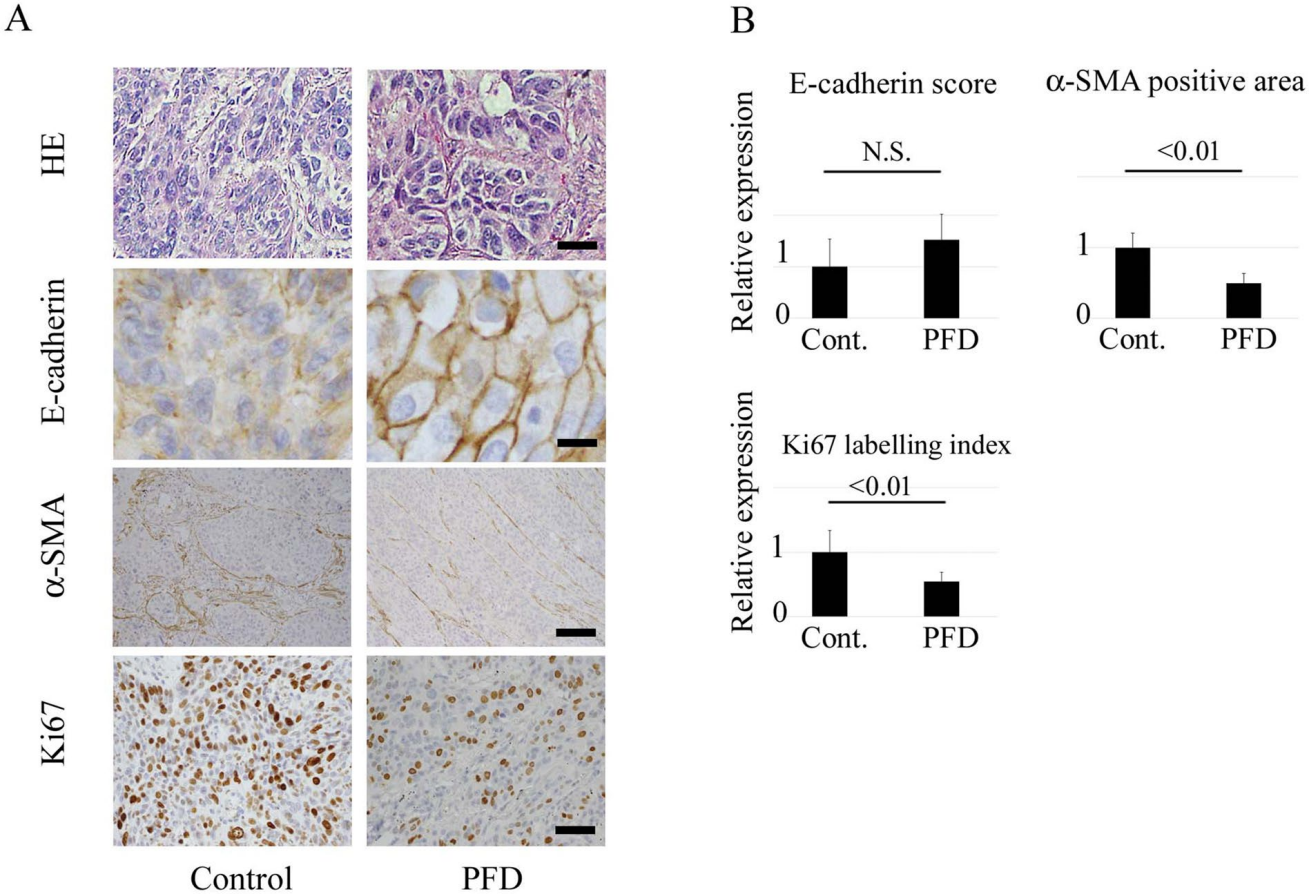
Effects of pirfenidone on cancer tissues from the non-small lung cancer patients with idiopathic interstitial pneumonia. (**A**) Representative findings of histological evaluation for hematoxylin–eosin staining, E-cadherin, and Ki67 of tumors obtained from human non-small cell lung cancer (NSCLC) with or without pirfenidone (PFD) treatment. Scale bar: 100 µm for hematoxylin–eosin staining, 50 µm for E-cadherin, and 200 µm for α-SMA and Ki67. (**B**) Panels show the scored expression of E-cadherin, the relative area of α-SMA expression, and the Ki67 labeling index as the means ± SD. Significance was tested with the Mann–Whitney U test.

In the crosstalk between cancer and stroma cells, two closely interactive pathways are established^18^: one is the “afferent” pathway where stroma cells affect cancer responses, and the other is the “efferent” pathway where cancer cells trigger a reactive response in the stroma. The present study demonstrated the “afferent” pathway and the effect of PFD on it. Then, to determine the effects of NSCLC cells on CAFs and the effect of PFD on the “efferent” pathway, the changes in CAFs with A549-CM that were or were not pretreated with PFD were further examined. A549-CM was prepared with the same method as noted above: in the process of CM preparation, PFD was added to the medium and removed after 24 h of treatment. A549-CM elongated the morphology of CAFs with higher expression of α-SMA, and PFD pretreatment decreased the changes in CAFs (Fig. 2F). A549-CM treatment enhanced the migration and invasion of CAFs, and these abilities were inhibited by pretreatment of A549 with PFD (Fig. 2G). Further, to assess the effects of PFD on interactions between CAFs or LNFs and NSCLC cells, a 3D co-culture model of CAFs or LNFs and A549 cells was established (Sup. Figure 5). Co-culture with CAFs or LNFs changed the layers of A549 cells from single to multiple, and more invasive activity was observed in CAF-CM-treated cells than in LNF-CM-treated cells. The layers of A549 cells became thinner, and invasiveness was also decreased with co-treatment with PFD. These data suggest that CAFs and LNFs have a strong effect on the EMT status of NSCLC cells, and PFD suppressed the tumor-stromal interaction, in addition to the direct effect on fibroblasts or NSCLC cells.

### Inhibitory effect of PFD in an in vivo model of NSCLC cells and CAFs

To examine the effects of PFD on tumor progression in vivo, A549 or NCI-H358 cells with CAFs at a 2:1 ratio were transplanted into the right side of the back, and cells without CAFs were transplanted into the left side of the back subcutaneously into nude mice. As shown in Fig. 2, A549 cells were transplanted with CAFs from cell type #3 (Fig. 1), while NCI-H358 cells were transplanted with cell type #2. Two weeks after implantation, daily administration of PFD was started and continued for three weeks. Tumor growth was greater in the CAF co-implantation group, and PFD significantly inhibited tumor growth in A549 cells, whereas the difference in tumor growth was not significant in NCI-H358 cells (Fig. 3A). PFD also showed a tumor growth inhibitory tendency in the NSCLC alone group in both cell types. Immunohistological analysis showed that the expression of E-cadherin was lower in the co-implantation group than in the control group (0.27 times in A549 cells, 0.37 times in NCI-H358 cells), but it was recovered by PFD administration (Fig. 3B, C). α-SMA expression showed more diffuse thick bundles in the co-implantation group than in the control group, and they became sparsely thin bundles with PFD treatment, both in the co-implantation and the NSCLC alone group, which was supported by the quantitation of the α-SMA-positive area. PFD also reduced the expression of the proliferation marker Ki67, with a significant decrease in the Ki67 labelling index. These findings suggest that CAFs promote tumor progression, and PFD suppresses tumor progression with an inhibitory effect on tumor-stroma crosstalk in vivo.

### Impact of PFD on human NSCLC tissues

To determine the impact of PFD on human NSCLC tissues, E-cadherin, α-SMA, and Ki67 were evaluated in tumor specimens from NSCLC patients who were treated with PFD for their IPF. For the control, tumor specimens of NSCLC patients with IPF who were not administered PFD were also evaluated. The patient characteristics of both groups are summarized in Table 1. Most of the patients had squamous cell carcinoma (Table 1 and Fig. 4A). Although the expression of E-cadherin was nearly identical, the α-SMA-positive area and the Ki67 labelling index were decreased in tumors of patients treated with PFD (Fig. 4A, B). The median follow-up period was 3.7 years. Thus, PFD treatment for IPF could have affected tumor status in human tissues.

**Table 1:** Patients’ characteristics.

Variable	Control group N = 9	PFD group N = 9*
*Sex*		
Male/female	9/0	6/3
Age, y, median (range)	74.7 (67–82)	71.9 (60–82)
*Histological ILD type*		
UIP	5	7
DIP	1	0
Unknown	3	2
Cancer type*		
Adenocarcinoma	2	3
Squamous cell carcinoma	7	8
p-Stage*		
IA/IB/IIA/IIB/IIIA	0/3/4/1/1	7/2/0/1/1
*Operative method*		
Lobectomy	8	4
Segmentectomy	1	2
Wedge resection	0	3
*AE-ILD*		
Yes/no	1/8	1/8
Survival (months)	17.9	15.0
Outcome		
Alive	3	6
Alive with disease	1	0
Dead of cancer	3	2
Dead of respiratory failure	1	1
Dead of other diseases	1	0

*PFD* pirfenidone, *ILD* interstitial lung disease, *UIP* usual interstitial pneumonia, *DIP* desquamative interstitial pneumonia, *AE* adverse event.

*Two of 9 PFD-Group patients had double tumors.

## Discussion

In the present study, PFD was shown to attenuate fibroblast activity and inhibit tumor-stromal interactions. PFD significantly inhibited the activity of NHLFs in invasion and migration, as well as fibroblast activation markers such as α-SMA, releasing growth factors, extracellular matrix proteins, and angiogenic factors, consistent with other papers^8,10^. Of note, PFD inhibited both Smad2- and STAT3-phosphorylation (Sup. Figure 1G). TGF-β signaling is important to promote tumor growth in tumor-fibroblast interactions^19^, and IL-6 is also a common cytokine that enhances TGF-β signaling resulting in epithelial cell EMT and stimulates tumor progression^20^. While IL-6 and TGF-β signaling has been reported to be important for interaction between cancer cells and CAFs in a cancer microenvironment^1^, PFD inhibits both Smad2- and STAT3-phosphorylation, indicating that it may have synergistic potential for inhibition of both IL-6 and TGF-β signaling. Although PFD was found to have a mild effect in most of the assays in the present study, we believe that the effect is clinically significant.

Usually, CAFs are reported to be more activated than LNFs, showing higher α-SMA expression, collagen gel contraction, and producing relevant signal mediators including growth factors, cytokines, chemokines, and other immune modulators^21,22^. In addition, CAFs are reported to be more competent as tumor growth enhancers than LNFs in vivo^23^. As in these other studies, CAFs were more activated than LNFs in the present study, although there was only a small difference in some results. A clinical trial by Iwata et al. that used PFD in the perioperative period to prove its efficacy in preventing acute exacerbation of interstitial pneumonia demonstrated that PFD did not affect wound healing after surgical resection of lung cancer^24^. They also reported that lung tissues of PFD-treated patients were less damaged, with lower scores of IPF features than control patients^25^. Thus, PFD could decrease the activation of both LNFs and CAFs, so that the difference between CAFs and LNFs decreased.

In the present study, tumor volume was significantly higher with the lower E-cadherin expression and higher α-SMA expression in the CAFs co-implantation group than in the control group in vivo. These results are consistent with the changes in NSCLC cells co-cultured with CAF or LNF-CM in vitro*,* as seen in Fig. 2. Thus, the tumor aggressiveness in the CAFs co-implantation group may be enhanced by EMT changes induced by CAFs. We previously reported that tumor volume was not significantly different between control and PFD-administered groups^15^, as well as in the present study. In the present experiments, PFD significantly inhibited tumor growth by A549 cells in the CAF co-implantation group. Thus, when considering the effects of PFD, it might inhibit tumor growth more effectively by targeting tumor-stroma crosstalk interaction than by targeting the tumor itself, as described in previous studies^13,26^. In a previous study, Mediavilla-Varela et al. found no significant difference in regard to the growth rate of tumors in untreated and PFD-treated mice using an in vitro co-culture model, which is in contrast to the present results^12^. Several reasons for this difference are possible, including the different cell composition ratios, absolute cell numbers, and origin of CAFs. Whereas researchers have transplanted CAFs and cancer cells at various concentrations from 1:0.5 to 1:10 in previous reports, a 1:2 ratio was chosen to avoid too much of an impact of CAFs on tumor formation^12,13,27^. There may be a suitable proportion for tumor growth in regard to CAFs and cancer cell composition, and CAF activity itself may differ depending on the origin, resulting in a different degree of tumor growth.

EMT status and activation of CAFs in the tumor specimens from the patients who were administered PFD for their IPF before surgical resection were also evaluated, and they were compared with those from patients who were not administered PFD in the same period. Although the mean administration period was just less than a month, histological changes were seen in expression of α-SMA in tumor stroma, as well as EMT or proliferation markers of cancer cells, suggesting that PFD administration might inhibit tumor-stroma interactions and tumor growth in the human body.

This study has some limitations, including the ability to generalize the results to the entire spectrum of NSCLC. Two different lung adenocarcinoma cell lines were used, since they are well known to be responsive to EMT. An attempt was made to verify whether EMT also occurs in squamous cell carcinomas, but typical EMT was not induced by TGF-β. As Gabasa et al. reported, there may be different mechanisms involved in TGF-β signaling, as well as aberrant CAF-carcinoma crosstalk in adenocarcinomas and squamous cell carcinomas^28^. The other was the special patient group, since only patients with IPF were included. All patients included in this study had IPF with lung cancer and received PFD to prevent acute exacerbations of interstitial pneumonia. However, surgical resection is unlikely to be performed in lung cancer patients with IPF because of their reduced respiratory function, even when the clinical stage score is low. Though an attempt was made to locate several cases from the 25 institutions in our group, the records of only 9 cases could be obtained. Thus, additional research is needed to fully understand the role of PFD for lung cancer patients in clinical settings with a greater number of samples from IPF patients.

Recently, Vancheri et al. reported that lung cancer and IPF share many common pathogenic pathways^29^. King et al. also reported that pulmonary tissues have in common an abundant stiff desmoplastic stroma rich in collagen and activated fibroblasts/myofibroblasts^30^. Furthermore, it is well known that the incidence of lung cancer is higher in IPF patients; thus, the tumors of IPF patients may have special characteristics when compared with the tumors that occur in normal lungs, and PFD might have worked better in the present study setting. Miura et al. reported that the incidence of lung cancer in IPF patients was significantly lower in the PFD-treated group than in the non-PFD-treated group^31^. These results suggest that PFD might attenuate fibroblast activation in IPF lungs, resulting in inhibition of carcinogenesis, as well as tumor progression. There was no significant difference in survival between the PFD-treated group and the non-PFD-treated group in the small sample of the present study. Thus, larger prospective clinical investigations, preferably including patients with normal lungs, will be necessary to clarify the anti-tumor effect of PFD in NSCLC patients. Nintedanib is another anti-fibrotic drug of multiple tyrosine kinases for IPF that has already been approved as a treatment for advanced lung adenocarcinoma combined with the cytotoxic agent docetaxel. Gabasa et al. performed experiments similar to the present one using nintedanib instead of PFD and reported that nintedanib abrogated the stimulation of growth and invasion in a panel of carcinoma cell lines induced by secreted factors from activated CAFs in adenocarcinoma^28^. Although PFD works differently than nintedanib, PFD may also be used as an anticancer agent.

In conclusion, the present study showed that PFD might inhibit not only fibroblast activity, but also the crosstalk between cancer cells and fibroblasts. Together with the previous report of its effect on EMT inhibition, PFD may have great potential as a novel treatment for NSCLC that acts from multiple perspectives.

## Materials and methods

### Cell lines

A549, NCI-H358, and NCI-H520 cells were purchased from the American Type Culture Collection (ATCC, Manassas, VA, USA) and maintained in Roswell Park Memorial Institute (RPMI) 1,640 medium with 10% fetal bovine serum (FBS) and 100 U/mL penicillin/streptomycin (Wako Pure Chemical Industries, Ltd., Osaka, Japan) in a humidified incubator of 5% CO_2_ at 37 °C. Normal human lung fibroblasts (NHLFs) were purchased from Lonza (Walkersville, MD, USA) and maintained using a Fibroblast Growth Media Kit (FGM) (Lonza, Basel, Switzerland) under the same conditions.

### Primary culture of fibroblasts

Primary human fibroblasts were isolated from surgically explanted patient lungs. The fibroblasts from the non-necrotic parts of the tumors were defined as carcinoma-associated fibroblasts (CAFs), and those from normal lung were defined as lung normal fibroblasts (LNFs). Briefly, cancerous tissue or normal lung far from cancerous tissue was obtained aseptically from three patients with NSCLC undergoing pulmonary resection. The Institutional Review Board for Clinical Research at Osaka University Hospital (Osaka, Japan) approved the study protocol, and written, informed consent for surgical intervention was obtained from each patient. Tissues were digested for 6 h in 1 mg/mL collagenase I (Sigma-Aldrich, St. Louis, MO, USA), and the cells were then plated in Dulbecco’s modified Eagle’s medium (DMEM) (Sigma-Aldrich) containing 10% FBS. Proliferated fibroblasts were confirmed by immunohistochemistry, as described^1^. All experiments were performed in triplicate using pairs of primary cultured CAFs and LNFs between passages 3 and 9.

### Production of conditioned media of primary fibroblasts and NSCLC cells

For co-culture, conditioned media (CM) were obtained from NSCLC cells and primary cultured LNFs and CAFs. Semi-confluent (70%) A549 cells and nearly 100% confluent LNFs/CAFs were each cultured in DMEM with 250 μg/mL PFD for 24 h. Then, the medium was replaced with DMEM containing 0.1% BSA modified to be free of PFD, and the culture was continued for another 24-h. The resulting CM were filtered with a Minisart Syringe Filter 0.45 μm (Sartorius, Goettingen, Germany) and used when freshly collected or stored at −80 °C until use.

### Antibodies and reagents

Antibodies used for Western blotting and immunofluorescence were as follows: anti-α-SMA (ab5694; Abcam, Cambridge, England), anti-collagen Type I (600-401-103-0.1; Rockland, Inc., PA, USA), anti-fibrinogen (ab23750, Abcam), anti-Periostin (ab14041, Abcam), anti-Smad2 (#3103S; Cell Signaling, Beverly, MA, USA), anti-phosphorylated Smad2 (#3101S; Cell Signaling), anti-Stat3 (#9139S; Cell Signaling), and anti-phosphorylated Stat3 (#9145S, Cell Signaling). Antibodies for the immunohistochemistry experiments were as follows: anti-E-cadherin mAb (M3612; Dako, Carpinteria, CA, USA), anti-α-SMA (ab5694; Abcam), and anti-Ki67 (M7240; Dako). Transforming growth factor (TGF)-β1 was purchased from R&D Systems (240-B; Minneapolis, MN, USA). For in vitro studies, PFD was provided by Shionogi & Co., Ltd. (Osaka, Japan) and dissolved in DMSO to a concentration of 50 μg/mL. For in vivo studies, Pirespa tablets were purchased from Shionogi & Co. and dissolved in DMSO, using one-fifth of the final volume of the total solvent, after crushing using a micro smash (TOMY SEIKO CO., LTD, Tokyo, Japan). Sterile normal saline at four-fifths of the final total volume was then added to bring PFD to a final concentration of 40 mg/mL.

### RNA extraction and real-time RT-PCR

Cells were treated under the indicated conditions, and then total RNA was extracted using an RNeasy Mini Kit (Qiagen, Tokyo, Japan). Real-time RT-PCR (α-SMA, Hs00426835_g1; Collagen I, Hs00164004_m1; VEGF, Hs00900055_m1; IL-6, Hs00985639_m1; HAS-2, Hs00193435_m1 (Applied Biosystems, Tokyo, Japan)) was performed using a CFX96 system (Bio-Rad Laboratories, Inc., Hercules, CA, USA), and relative expression levels were calculated by the comparative Ct method. All experiments were performed in duplicate, and each experiment was performed three times independently. Data shown are the averages of these experiments.

### SDS-PAGE and western blot analysis

Monolayers of cultured cells were treated under the indicated conditions, and proteins were extracted with RIPA buffer (#9,806, Cell Signaling). Cell extracts were resolved with sodium dodecyl sulfate polyacrylamide gel electrophoresis (SDS-PAGE) and immunoblotted as described^32^. Western blotting after extraction of membrane proteins was performed using a Cell Surface Protein Isolation Kit (P74008, Takara, Tokyo, Japan) according to the manufacturer’s protocol. Quantification of immunoblots was performed by Image Lab software as per the manufacturer’s instructions (Bio-Rad Laboratories, Inc.). All experiments were performed two times independently. Data shown are representative experiments and their quantitative values.

### Trans-well motility assays (migration assays)

A total of 1 × 10^6^ cells, which were suspended in DMEM containing 1% FBS (NHLFs in Fig. 1) or serum-free DMEM (A549, NCI-H358, and CAFs in Fig. 3 and Sup. Figure 1), were plated in the upper chamber of a transwell with polyethylene terephthalate membranes (pore size 8 µm; Becton Dickinson, Franklin Lakes, NJ, USA). In the lower chamber, FGM with or without TGF-β or PFD (NHLFs; Fig. 1) or DMEM containing 0.1% BSA or CM with or without PFD pretreatment (NSCLC cells and CAFs; Fig. 3 and Sup. Figure 1) was added. The cells were stained with hematoxylin, and the number of cells on the lower side of the filter was counted under a microscope after incubation for 24 h. Data were collected from three independently performed experiments.

### Gel contraction assays

NHLFs were cast into collagen gels according to the manufacturer’s protocol (Thermo Fisher Scientific Inc., Waltham, MA, USA). Briefly, collagen gel was prepared with Collagen I, Rat Tail (Thermo Fisher Scientific Inc.), 1 N sodium hydroxide, phosphate buffered saline (PBS), and sterile distilled water so that the final collagen concentration was 1.5 mg/mL. NHLFs cells (2 × 10^5^ cells/mL) suspended in FGM were mixed with collagen gel and dispensed into each well of 24-well tissue culture plates. After collagen polymerization, cultures were incubated for 3 days with or without TGF-β and PFD. Gel contraction was initiated by detaching the perimeter of the collagen gels from the side of the wells. The collagen gel area was determined 24 h after detaching using Image J. Data were collected from three independently performed experiments.

### Enzyme-Linked Immunosorbent Assay (ELISA) for TGF-β1 and IL-6

Cells were seeded in 6-well plates (5 × 10^4^ cells/well). After 2 days of incubation, TGF-β1 was added to the medium. On the next day, TGF-β1-containing medium was removed, and the cells were fed with serum-free DMEM (800 μl), with or without PFD. For LNFs or CAFs in Fig. 2, addition and removal of TGF-β1 was not performed. Conditioned media were collected after 24 h, and TGF-β1 or IL-6 was quantified using an ELISA according to the manufacturer’s protocol (Human Quantikine; R&D Systems). All experiments were performed in duplicate, and each experiment was performed three times independently. Data shown are the averages of these experiments.

### Wound healing assays

NSCLC cells were seeded in 6-well plates (1 × 10^6^ cells/well). After the formation of a confluent monolayer, cells were scratched with a pipette tip. Culture medium was immediately removed and replaced by DMEM containing 0.1% BSA with or without PFD or LNF/CAF-CM with or without pretreatment of PFD. After 24 h of incubation, the remaining area after quitting the cell migration into the wound was measured, and quantification of the remaining area was performed using Image J. Data were collected from three independently performed experiments.

### Invasion assays

Transwell chambers with 8-μm pores (Becton Dickinson) were coated with 100 μg/mL Matrigel (Becton Dickinson). Cells (1 × 10^6^ cells/well) suspended in serum-free DMEM were plated in the Matrigel-coated upper chamber. In the lower chamber, DMEM containing 0.1% BSA with or without PFD or CM with or without PFD pretreatment was added. After incubation for 24 h, cells were stained with Hoechst 33342, and the number of cells on the lower side of the filter was counted under a microscope. Data were collected from three independently performed experiments.

### Animal studies

To analyze the effects of PFD on NSCLC and CAFs in vivo, A549 (1 × 10^6^ cells/each) suspended in 100 μL serum-free DMEM with or without CAFs (5 × 10^5^ cells) was injected subcutaneously into the left and right sides of the back of 4-week-old female nude mice. The mice were obtained from CLEA Japan, Inc. (Tokyo, Japan). Two weeks after implantation, the mice were divided into 2 groups and intraperitoneally administered either 200 mg/kg PFD or sterile water as a control for 3 weeks, following our previous report on the dose and method of administration^15^. Mice were monitored weekly for subcutaneous tumors. Tumor volume (TV) was calculated according to the formula: TV (cm^3^) = d^2^ × D/2, where d and D were the shortest and the longest diameters, respectively. All tumor tissues were harvested and processed for morphological and immunohistochemical analyses. All animal studies were approved by the Osaka University Animal Experiment Committee (Approval No. 28–007-009). All experiments were performed in accordance with the Osaka University Regulations on Animal Experiments.

### Immunohistochemistry

Immunohistochemistry was performed as previously described^33^. All sections stained with E-cadherin were scored in a semiquantitative manner to reflect both the intensity and percentage of cells. The staining intensity was classified as 0 (no staining), + 1 (weak staining), + 2 (moderate staining), or + 3 (strong staining) and multiplied by the percentage of positive cells. Smooth muscle actin (SMA) staining was determined as area stained positive by total dyed area (SMA-positive area/total dyed area). The labeling index (labeling frequency %) of Ki67 staining was calculated with the following formula: (number of positive nuclei/total number of cells) × 100. All quantities or cell counts were calculated using CellSens (Olympus Imaging Corp., Tokyo, Japan). All immunohistochemistry (IHC) results were quantified by counting more than 200 tumor cells in five randomly selected areas per specimen.

### Patients

Tumor specimens from 9 NSCLC patients who were administered PFD for their IPF before surgical resection were evaluated for E-cadherin, SMA, and Ki67. For control, tumor specimens of 9 NSCLC patients with IPF who were not administered PFD were also evaluated. The clinical data were retrospectively obtained from medical charts, and the patients’ characteristics are summarized in Table 1. All patients treated with PFD started administration before surgery in expectation of preventing acute exacerbations of IPF, and the mean period of administration before surgery was 26.2 ± 12.0 (range, 11–53) days. PFD at a dose of 600 mg/day was administered orally to patients for the first 2 weeks, and the dose was then increased to 1,200 mg/day for the next 2–4 weeks preoperatively. The administration dose and dose-increase interval were established according to the protocol included in the Japanese PFD package insert. The final administration dose was finally decided by the attending physician considering each patient’s condition; thus, the final administration dose was 600 mg per day in 4 of 9 PFD-treated patients and 1,200 mg in 5 of 9 PFD-treated patients. Two of 9 PFD-treated patients had double cancers. Survival time was defined as the time from surgery to the date of death or the last follow-up date. Patients treated with PFD came from institutions that belonged to the Thoracic Surgery Study Group of Osaka University (TSSGO), and the control group consisted of patients from Osaka University Hospital. The institutional review board of each institution approved this study (approval no. 15341). Written, informed consent was obtained from all patients for use of patient information and clinical samples in the present study. Written, informed consent was also obtained from former patients, who were given the right to revoke consent and opt out from this investigation. This study was performed in accordance with relevant laws and regulations.

### Statistical design and data analysis

The chi-squared test, Mann–Whitney U test, and repeated measures ANOVA were used to compare the results, as appropriate; a *p* value < 0.05 was considered significant.

## Supplementary information

Supplementary file1 (DOCX 3516 kb)

## Data Availability

All data generated or analyzed in this study are included in this published article.
