# Supplementary material for: Effects of pirfenidone targeting the tumor microenvironment and tumor-stroma interaction as a novel treatment for non-small cell lung cancer
Source: Sci Rep. 2020 Jul 2;10:10900. doi: 10.1038/s41598-020-67904-8 (PMC7331721; doi:10.1038/s41598-020-67904-8)
Supplement: Supplementary file 1 — Supplementary file1 (DOCX 3516 kb) [file 41598_2020_67904_MOESM1_ESM.docx]

Effects of pirfenidone targeting the tumor microenvironment and tumor-stroma interaction as a novel treatment for non-small cell lung cancer

**Authors**

Ayako Fujiwara, Soichiro Funaki, Eriko Fukui, Kenji Kimura, Takashi Kanou, Naoko Ose, Masato Minami, and Yasushi Shintani.

**Supplementary information**

Supplemental Figure 1.

Inhibitory effects of pirfenidone on myofibroblast differentiation and activation in normal human lung fibroblasts.


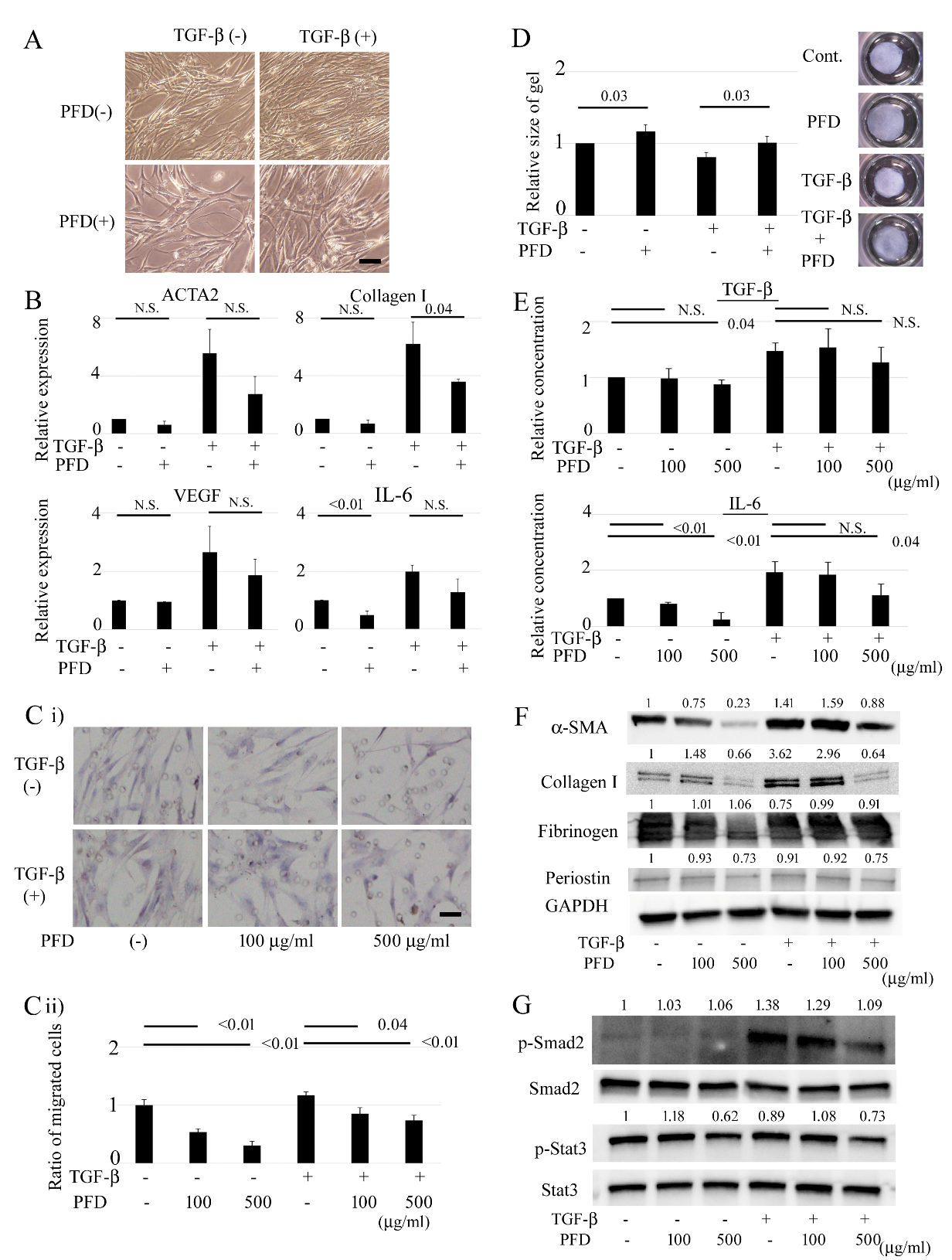


A. Normal human lung fibroblasts (NHLFs) were treated with DMSO (control), pirfenidone (PFD, 500 μg/mL), transforming growth factor β1 (TGF-β1) (0.5 ng/mL), or PFD in addition to TGF-β1 for 2 days. Phase contrast pictures were taken. Scale bar: 200 µm. When the NHLFs are incubated with TGF-β1, they show a more slender, elongated shape under phase-contrast microscopy. By co-treatment with PFD, NHLFs become thicker and shorter, both in TGF-β1-treated and non-treated cells.

B. NHLFs are treated with DMSO, PFD, TGF-β1, or PFD, in addition to TGF-β1 for 24 h. Subsequently, total RNA is extracted, and real-time RT-PCR is performed to detect ACTA2, Collagen I, VEGF, and IL-6 mRNA. All experiments were performed three times independently with duplicates, and the results are the means ± SD. Significance was tested with the Mann-Whitney U test. The mRNA levels of ACTA2 and collagen I are upregulated by TGF-β1 and suppressed by co-treatment with PFD. PFD also suppresses the changes in expression of the pro-inflammatory cytokine IL-6 induced by TGF-β1, but not in the expression of the potent angiogenic factor VEGF.

C. Motility assays are performed, and cells traversing the filter are counted. DMSO, PFD (100 or 500 µg/mL), TGF-β1 (0.5 ng/mL), or PFD plus TGF-β1 is added to the lower side of the chamber. Panel i) shows representative findings of NHLFs. Scale bar: 200 µm. Panel ii) shows the ratio of migrated cells compared to control. All experiments were performed three times independently, and the columns represent the means ± SD. TGF-β1 induces upregulation of cell migration, which is suppressed by PFD in a dose-dependent manner.

D. NHLFs are treated with DMSO, PFD (500 µg/mL), TGF-β1 (0.5 ng/mL), or PFD in addition to TGF-β1 for 72 h in collagen gels. After detaching the collagen gels, another 24-h incubation is performed, and the collagen gel area is determined. All experiments were performed three times independently, and the pictures shown are representative experiments. The results are the means ± SD. Significance was tested with the Mann-Whitney U test. TGF-β1 enhances gel contraction, which is suppressed by 500 μg/mL PFD.

E. NHLFs are treated as in A. The medium is changed to FBS-free medium, and TGF-β1 or IL-6 in the conditioned medium is then measured 24 h after the medium change by ELISA. All experiments were performed three times independently with duplicates, and the results are the means ± SD. Significance was tested with the Mann-Whitney U test. Although the suppressive effect of low-concentration (100 μg/mL) PFD is small, 500 μg/mL PFD suppresses both TGF-β1 and IL-6 supernatant concentrations.

F. NHLFs are treated as in A and then analyzed by Western blotting for α-SMA, collagen I, fibrinogen, periostin, and GAPDH (loading control). PFD inhibits myofibroblast differentiation, although the effect of 100 μg/mL PFD is small. Data shown are representative experiments, and quantification was performed.

G. NHLFs are treated with DMSO, PFD (100 or 500 μg/mL), TGF-β1, or PFD plus TGF-β1. PFD is added to the medium a day earlier than TGF-β1. The cells are treated with TGF-β1 for 30 min and then analyzed by Western blotting for phospho (p)-Smad2, Smad2, p-Stat3, and Stat3. All experiments were performed two times independently. Data shown are representative experiments and their quantitative values. PFD at 500 μg/mL inhibits both Smad2- and STAT3-phosphorylation.

Supplemental Figure 2.

Inhibitory effects of pirfenidone on EMT status of adenocaricinoma cells in response to conditioned media from cancer-associated fibroblasts or lung normal fibroblasts.


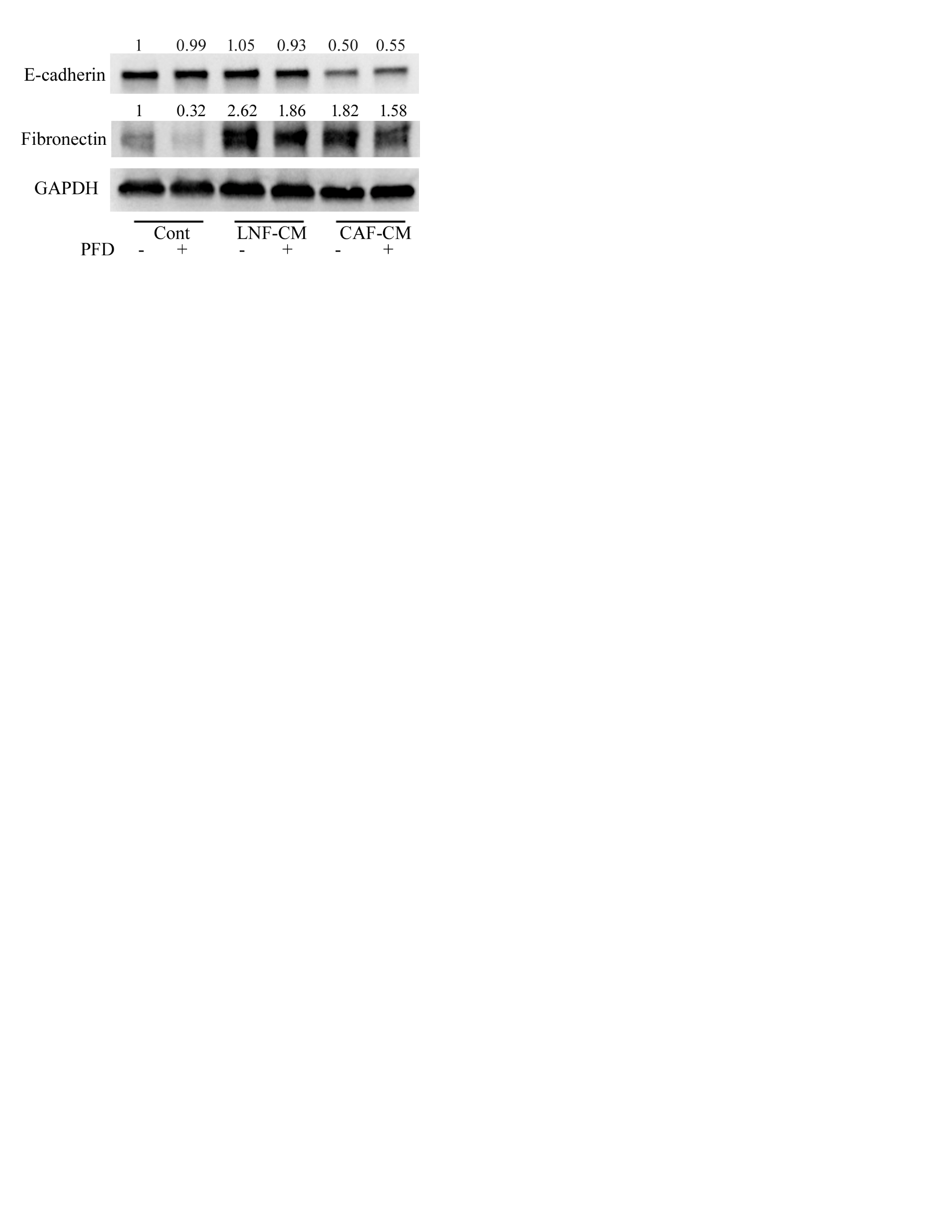


A549 cells are cultured with CM from LNFs or CAFs for 2 days and compared to control by Western blotting for E-cadherin, fibronectin, and GAPDH (loading control). In the process of CM preparation, LNFs or CAFs are cultured with or without PFD for 24 h, and then PFD is removed. All experiments were performed two times independently. Data shown are representative experiments and their quantitative values.

Supplemental Figure 3.

Inhibitory effects of pirfenidone on the interaction between primary cultured fibroblasts and non-small cell lung cancer cells.


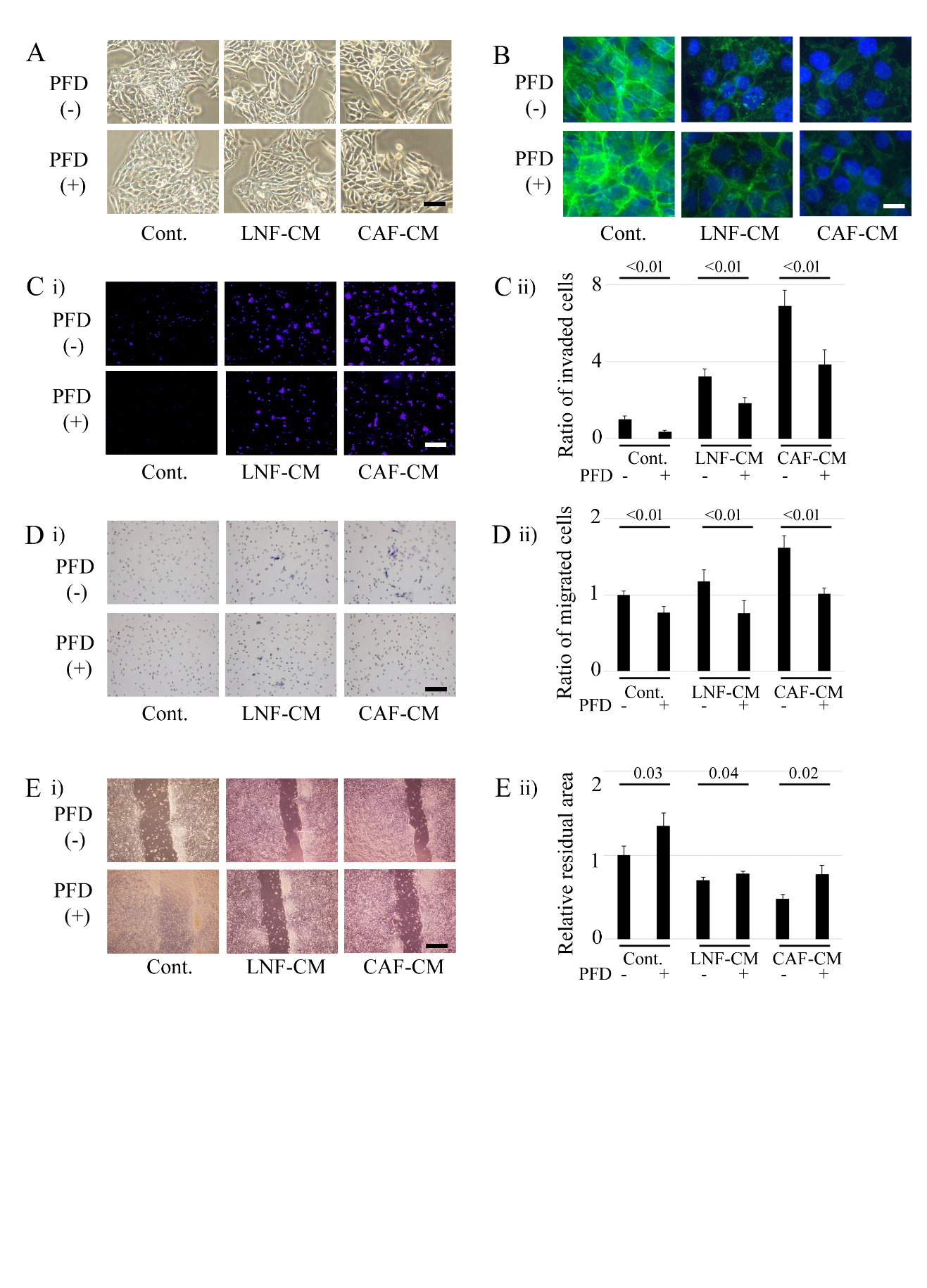


A. NCI-H358 cells are cultured with the same method described in the legend of Figure 3A. Scale bar: 100 µm.

B. NCI-H358 cells are treated as in A, and the cells are then immunofluorescently stained for E-cadherin. Scale bar: 50 µm.

C. Invasion assays are performed as noted in the legend of Figure 3C. Panel i) Scale bar: 200 µm.

D. Motility assays are performed as noted in the legend of Figure 3D. Panel i) Scale bar: 130 µm.

E. Wound healing assays are performed as noted in the legend of Figure 3E. Panel i) Scale bar: 400 µm.

F. Hematoxylin-eosin stain of cross sections of 3D gel co-cultures of A549 cells and LNFs or CAFs with or without pirfenidone. Arrows indicate cell invasion. Scale bar: 130 µm. Scale bar: 400 µm.

Supplemental Figure 4.

Inhibitory effects of pirfenidone on EMT status of squamous cell carcinoma cells in response to conditioned media from cancer-associated fibroblasts or lung normal fibroblasts.


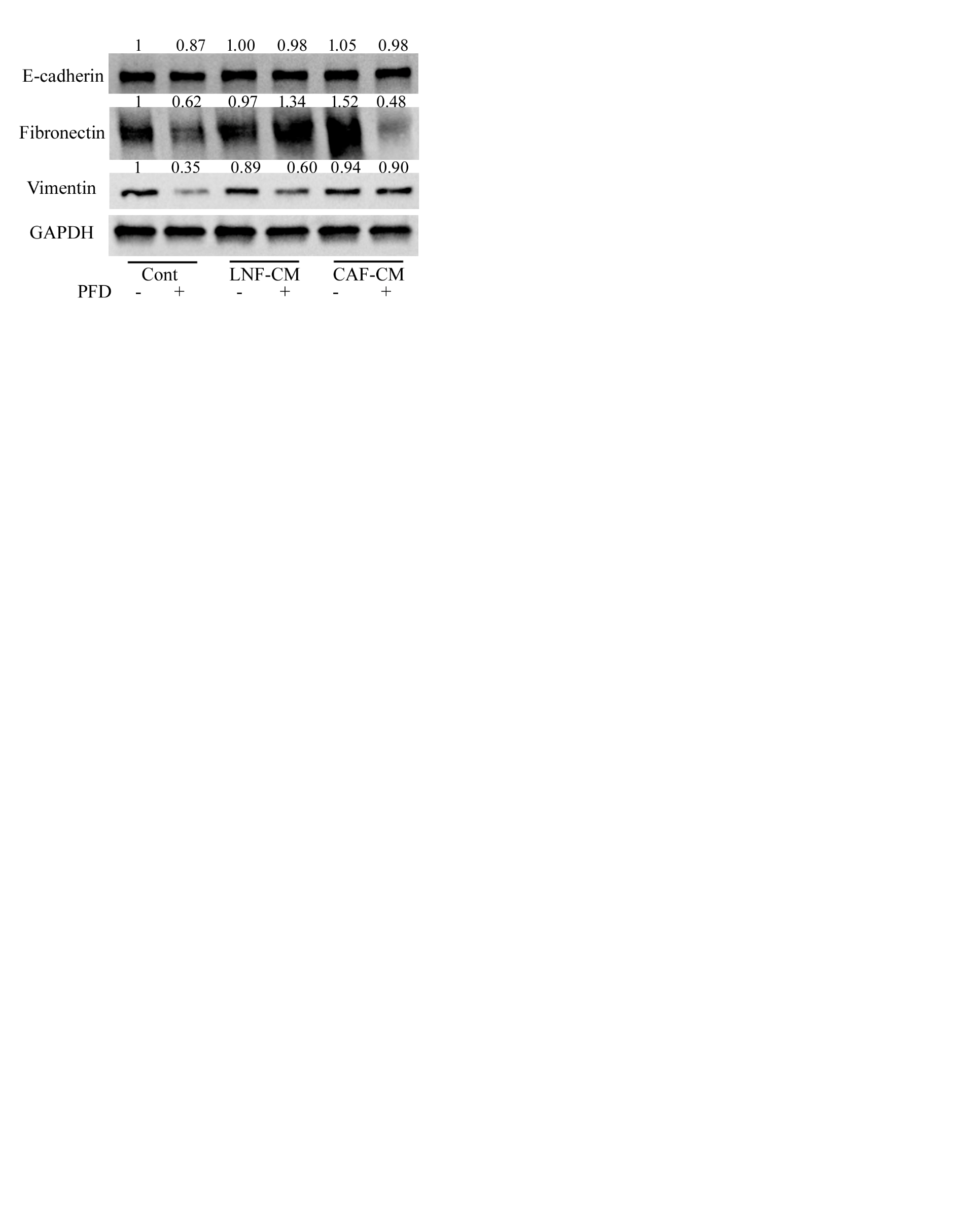


NCI-H520 cells are cultured with CM from LNFs or CAFs for 2 days and compared to control by Western blotting for E-cadherin, fibronectin, vimentin, and GAPDH (loading control). In the process of CM preparation, LNFs or CAFs are cultured with or without PFD for 24 h, and then PFD is removed. All experiments were performed two times independently. Data shown are representative experiments and their quantitative values.

Supplemental Figure 5.

Inhibitory effects of pirfenidone on interaction between cancer-associated fibroblasts or lung normal fibroblasts and non-small cell lung cancer cells in three-dimensional gel co-cultures.


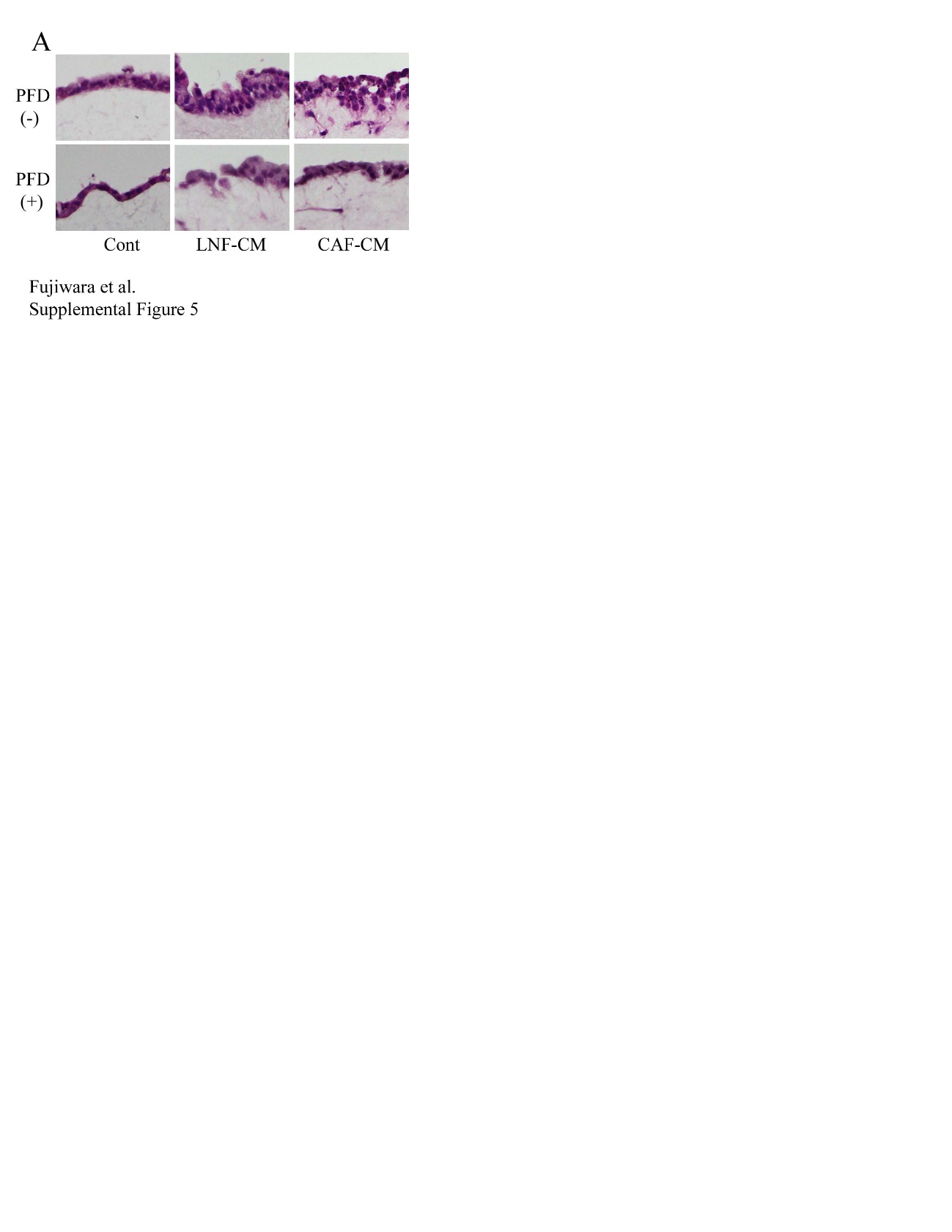


Three-dimensional (3D) gel co-cultures were carried out according to the manufacturer’s protocol (Nitta Gelatin, Tokyo, Japan). Briefly, collagen gels were prepared by mixing type I collagen (0.3% Cell matrix type IA; Nitta Gelatin) diluted with hydrochloric acid, 5× DMEM and reconstitution buffer with a ratio of 7:2:1 so that the final collagen concentration was 0.21%. First, the mixture (500μl/well) was cast into each well of a 24-well culture plate and allowed to polymerize at 37 °C for 30 min to make a base layer. Then, 500μl cell suspension of cancer-associated fibroblast (CAFs) or lung normal fibroblasts (LNFs) (5 × 10^4^ cells/well) in gels were dispensed on base layer and incubated at 37 °C for 30 min to make a top layer. For co-culture, A549 cells (1 × 10^5^ cells/well) were seeded on the surface of each gel and cultured in DMEM with or without pirfenidone (PFD). After incubation for 18 days with every 3 days of medium replacement, each gel was detached and cultured for additional 2 days. After 20 days of culture in total, the gel was fixed in formalin solution and embedded in paraffin, and vertical sections (4 μm) were stained with hematoxylin and eosin (HE).

HE stain of cross-sections of 3D gel co-cultures of A549 cells and LNFs or CAFs with or without PFD. Scale bar: 130 µm.
